# Hemizygous nonsense variant in the moesin gene *(MSN)* leads to a new autoimmune phenotype of Immunodeficiency 50

**DOI:** 10.3389/fimmu.2022.919411

**Published:** 2022-09-01

**Authors:** András L. Kovács, Judit Kárteszi, Zoltán Prohászka, Tibor Kalmár, Gábor Késmárky, Katalin Koltai, Zsuzsanna Nagy, Judit Sebők, Tibor Vas, Krisztián Molnár, Tímea Berki, Katalin Böröcz, Csaba Gyömörei, József Szalma, Miklós Egyed, Szabina Horváth, Péter Oláh, Dorottya Csuka, Viktória Németh, Rolland Gyulai

**Affiliations:** ^1^ Department of Dermatology, Venereology and Oncodermatology, Medical School, Clinical Centre, University of Pécs, Pécs, Hungary; ^2^ Genetic Counseling, Saint Rafael Hospital of Zala County, Zalaegerszeg, Hungary; ^3^ Research Group for Immunology and Haematology, Eötvös Loránd Research Network (Office for Supported Research Groups), Semmelweis University, Budapest, Hungary; ^4^ Genetic Diagnostic Laboratory, Department of Pediatrics and Pedriatic Health Center, Albert Szent-Györgyi Health Centre, University of Szeged, Szeged, Hungary; ^5^ Division of Angiology, 1st Department of Internal Medicine, Medical School, Clinical Centre, University of Pécs, Pécs, Hungary; ^6^ Nephrological and Diabetological Center, 2nd Department of Internal Medicine, Medical School, Clinical Centre, University of Pécs, Pécs, Hungary; ^7^ Department of Medical Imaging, Medical School, Clinical Centre, University of Pécs, Pécs, Hungary; ^8^ Department of Immunology and Biotechnology, Medical School, Clinical Centre, University of Pécs, Pécs, Hungary; ^9^ Department of Pathology, Medical School, Clinical Centre, University of Pécs, Pécs, Hungary; ^10^ Oral and Maxillofacial Surgery, Department of Dentistry, Medical School, Clinical Centre, University of Pécs, Pécs, Hungary; ^11^ Department of Hematology, Somogy County Mór Kaposi General Hospital, Kaposvár, Hungary; ^12^ Department of Dermatology, Medical Faculty, University Hospital Düsseldorf, Heinrich-Heine-University Düsseldorf, Düsseldorf, Germany

**Keywords:** juvenile loss of teeth, chronic leg ulcer, autoimmunity, whole exome sequencing, moesin protein, antiphospholipid syndrome, thyroiditis

## Abstract

Here, we present the findings of an investigation involving two male siblings with juvenile total tooth loss, early-onset chronic leg ulcers, and autoimmune thyroiditis, as well as focal segmental glomerulosclerosis with associated pulmonary emphysema in one and diabetes mellitus in the other. The clinical picture and lupus anticoagulant, cryoglobulin, and cold agglutinin positivity suggested the diagnosis of antiphospholipid syndrome. Flow cytometry analysis showed immunophenotypes consistent with immune dysregulation: a low number of naive T cells, elevated CD4^+^ T cell counts, and decreased CD8^+^ T-cell counts were detected, and more than half of the T-helper population was activated. Considering the siblings’ almost identical clinical phenotype, the genetic alteration was suspected in the background of the immunodeficiency. Whole exome sequencing identified a previously not described hemizygous nonsense variant (c.650G>A, p.W217X) within exon 6 of the moesin (*MSN*) gene localized on chromosome X, resulting in significantly decreased *MSN* mRNA expression compared to healthy controls. We present a putative new autoimmune phenotype of Immunodeficiency 50 (MIM300988) characterized by antiphospholipid syndrome, Hashimoto’s thyroiditis, leg ulcers, and juvenile tooth loss, associated with W217X mutation of the MSN gene.

## Introduction

Primary immunodeficiency diseases (PIDs) are a diverse group of mostly inherited genetic disorders characterized by loss of immune function and predisposition to recurrent infections or other immune diseases, such as autoimmunity and lymphoid malignancies. Loss-of-function mutations in genes encoding cytoskeletal proteins could result in PIDs (1).

Ezrin, radixin, and moesin (ERM) are three closely related proteins that link membrane-associated proteins directly to actin filaments in the cell cortex (2). ERM proteins organize the interface between the actin cytoskeleton and the plasma membrane and consequently play a central role in determining the structure and function of the plasma membrane and associated molecules, filopodia and microvilli, cellular morphology, adhesion, and epithelial integrity, while also affecting insulin and Rho signaling pathways (3). The ERM proteins are also known to play an important role in the formation of the immunological synapse (IS) (4). IS formation involves receptor–ligand pair clustering and intracellular signaling molecule recruitment, with coincident removal of other membrane proteins from the IS. MSN blocks inhibitory glycoproteins in the IS by concentrating these on distal poles, while EZR plays a role in the formation of signalosomes and the trafficking of signaling molecules (e.g., ZAP-70). Microfilament–membrane linkage is critical to this process. ERM proteins are also critical modulators of cortical architecture during highly dynamic cell behaviors, such as mitosis, migration, and junction remodeling (5). Indeed, ERM proteins regulate B- and T-cell activation through controlling B- and T-cell receptor dynamics, scaffolding protein assembly, and hence membrane-associated intracellular signaling (6). Two ERM members ezrin and moesin are highly expressed in lymphocytes (**Supplementary Figures S1A, B**), where chemokine activation or antigen presentation results in a rapid de- and rephosphorylation cycle, causing polarization and morphological changes. It was also described in a mouse model that excessive or deficient ERM-mediated crosslinking results in impaired lymphocyte homing (7). ERM proteins also play an important role in the regulation of B-cell receptor (BCR) signaling by undergoing antigen‐induced conformational inactivation to facilitate lipid raft coalescence and BCR microclustering (8). Importantly, MSN has also been implicated in the IL-15–dependent proliferation of CD8^+^ and CD4^+^ Tregs, while MSN deficiency results in lymphocyte egress from lymphoid organs, causing persistent lymphopenia in peripheral blood. Recent studies suggest that ERM proteins are also responsible for aberrant cellular responses in the presence of viral, bacterial, and fungal pathogens. The fact that ERM proteins are positive regulators of X4‐tropic human immunodeficiency virus‐1 (HIV‐1) infection corroborates this theorem (9).

The moesin protein-coding *MSN* gene is located on chromosome Xq12. Its pathogenic variants cause an X-linked recessive primary Immunodeficiency, termed Immunodeficiency 50 (IMD50). IMD50 is characterized by childhood recurrent bacterial respiratory, urinary, and gastrointestinal infections; complicated varicella-zoster; and extensive molluscum contagiosum virus infection (10). Typical laboratory findings are persistent lymphopenia, fluctuating neutropenia, decreased CD4^+^ and CD8^+^ cell and low CD45RA^+^ T cell counts, increased senescent CD8^+^ cell proportions, impaired T-cell proliferative responses and antibody formation, low levels of circulating NK and B cells, and hypogammaglobulinemia. The disorder does not affect overall survival. To the best of our knowledge, IMD50 has been described in nine cases so far (11, 12). Eight of them carried an identical missense mutation (c.511C>T, p.R171W). This missense variant causes MSN mRNA degradation and diminished protein expression, particularly in lymphocytes. One single individual carried a truncating variant caused by a premature stop codon (c.1657C>T, p.R533X), who did not suffer from the viral infections noted above. All patients were in satisfactory general condition at the examination; however, they required immunoglobulin therapy. No lethality has been reported in the literature.

Here, we present two male siblings with juvenile total tooth loss, early onset chronic leg ulcers and autoimmune thyroiditis, lupus anticoagulant, cryoglobulin, and cold agglutinin positivity, as well as immunophenotypes consistent with immune dysregulation, in whom whole exome sequencing identified a previously not described hemizygous nonsense variant (c.650G>A, p.W217X) of the MSN gene. Based on detailed clinical, laboratory, and genetic analyses, we suggest a new autoimmune phenotype of IMD50.

## Materials and methods

### Laser Doppler flowmetry, toe pressure, and toe-brachial index

Laser Doppler flowmetry (LDF) is a noninvasive method based on the Doppler effect to detect blood flow in nutritive and thermoregulatory capillaries of the skin. The LDF instrument (PeriFlux System 5000, Perimed, Stockholm, Sweden) uses optical fibers to carry and detect laser light (wavelength, 780 nm). The LDF probe was attached to the skin by a double-sided adhesive tape. The detected flux signal is expressed as a perfusion unit (PU). Following the baseline skin perfusion detection at the temperature of the skin, the heatable probe can be set at 44°C. Due to the heat provocation, capillaries situated under the probe vasodilate, which is proportional to the increase of perfusion unit. The percent change of baseline and heat-provoked PU can indicate the local reserve capacity of related capillaries, which can be deteriorated in case of endothelial dysfunction or ischemia.

### Transcutaneous partial oxygen pressure measurement

Transcutaneous partial oxygen pressure measurement (tcpO_2_) is a noninvasive electrochemical method to detect the oxygen concentration of tissues. Precalibrated Clark electrode (Tina TCM 4000 oximeter, Radiometer, Denmark) was positioned on cleaned, hairless skin at the second intercostal space of the anterior chest wall as a reference probe, at the leg close to the ulcer, and at the dorsum of the foot with a self-adhesive fixation ring that was filled by contact liquid. The sensors are made of an oxygen-permeable membrane and a platinum–silver electrode with phosphate-buffered solution between them. A steady state in the supine position of the index limb was obtained for 15 min with the heating of probes to 44°C to achieve maximal vasodilation. The polarizing voltage generates an electrical potential difference that is proportional to the partial oxygen pressure of the tissue. This value is displayed in millimeters of mercury (mmHg). TcpO_2_ value of >50 mmHg is considered physiologic, while tcpO_2_ of <30 mmHg is viewed as a threshold for the diagnosis of severe ischemia.

### Patient samples and cell preparations

Peripheral blood and/or skin samples from the two patients, their relatives, and healthy volunteers were harvested after the provision of written informed consent and in accordance with the Declaration of Helsinki. Peripheral blood mononuclear cells (PBMCs) were isolated using Ficoll-Paque Plus (GE Healthcare, Chicago, IL, USA) media for further analysis.

### Whole exome sequencing

Genomic DNA was extracted from PBMCs by standard protocol. For exome sequencing, a total of 200 ng of genomic DNA was used for library preparation and sequenced with the exome kit Agilent SureSelectXT All Exon V6 (Agilent Technologies, Santa Clara, CA, USA) on the Genome Sequencer Illumina (HiSeq, Illumina, San Diego, CA, USA) platform (parameters: paired-end run type, read length: 2 × 150 bp, 60× average on target coverage). The 150-bp paired reads were aligned to the GRCh37.75 human reference genome by Burrows Wheel Aligner (BWA v0.7.9a) software (13). The variants were called by the Genome Analysis Toolkit Haplotype Caller (GATK v3.5) (Broad Institute, Cambridge, MA, USA) best practice and annotated by SnpEff (14) and VariantStudio (Illumina, San Diego, Ca, USA) software. Variants obtained by exome sequencing were filtered based on severity and frequency against public variant databases including dbSNP (https://www.ncbi.nlm.nih.gov/snp/) (15), ClinVar (https://www.ncbi.nlm.nih.gov/clinvar/) (16), NHLBI GO Exome Sequencing Project (ESP), (http://evs.gs.washington.edu/EVS/), GnomAD (http://gnomad.broadinstitute.org), and HGMD (http://www.hgmd.cf.ac.uk) (17) databases and an in-house clinical exome database of 300 unrelated Hungarian samples. Sanger sequencing was performed to confirm the moesin variant.

### Quantitative RT-PCR

Total RNA was extracted from PBMC using the Direct-zol RNA Miniprep Kit (Zymo Research, Irvine, CA, USA). Reverse transcription was performed from 250 ng of total RNA using SuperScript RT enzyme with random hexamer primers (Thermo Fisher Scientific, Waltham, MA, USA). The expression level of moesin (Hs01085682_g1) was determined by RT-qPCR on QuantSudio™ 1 (Applied Biosystems, Waltham, MA, USA), and the samples were normalized to β-actin (Hs99999903_m1) mRNA level. The _ΔΔ_CT method was used to quantify the differences.

### Western blot

Total protein was extracted from PBMCs using M-PER™ buffer (Thermo Fisher Scientific, Waltham, MA, USA) supplemented with Halt™ Protease and Phosphatase Inhibitor Cocktail (Thermo Fisher Scientific, Waltham, MA, USA). Protein concentration was measured using Qubit™ Protein Assay Kit (Invitrogen, Waltham, MA, USA). A total of 10 µg of proteins per sample was separated on a 4%–15% precast gel (Bio-Rad, Hercules, CA, USA) and transferred to a nitrocellulose membrane. The staining was performed using the following antibodies: anti-moesin (NBP2-32875, Novus Biological), anti-β-actin (ab115777, Abcam) primary antibodies and HRP conjugated goat antimouse (12-349, Millipore), goat anti-rabbit (ab6721, Abcam) secondary antibodies, respectively. Detection of targeted protein was carried out with ECL (ImmunoCruz™, Santa Cruz Biotechnology, Dallas, TX, USA) using a chemiluminescence system (Syngene, Cambridge, UK).

### Cell proliferation assay

PBMCs were cultured in RPMI 1640 medium containing 10% FBS, 1% l-glutamine, and 1% antibiotic/antimycotic solution at a density of 10^5^ cells/well and were activated with 15 μg/ml phytohemagglutinin (PHA). For the control treatment, culture medium alone was added to the PBMC suspension. After, a 72-h incubation at 37°C in 5% CO_2_ 3-[4,5-dimethylthiazol-2-yl]-2,5 diphenyl tetrazolium bromide (MTT) assay was performed. The absorbance was measured at 540 nm with the ELISA reader. All experiments were performed in triplicate. Data were expressed as a percentage change in the proliferation of PHA-activated PBMCs compared to the proliferation of untreated PBMCs in each group.

### Flow cytometric detection of regulatory T-cell subpopulations

Regulatory T cells and their subgroups were determined with multiparametric flow cytometry using cell surface-specific anti-CD8-FITC (BD UCH-T4), anti-CD4-PerCP (BD SK3), and anti-CD25-APC (BD M-A251) antibodies, and for intracellular staining, PE-conjugated anti-Foxp3 (259D/C7, Becton Dickinson, Franklin Lakes, NJ, USA) antibody. Intracellular staining was performed using the Foxp3/Transcription Factor Staining Buffer Set (eBioscience), following the manufacturer’s instructions. The fluorescence of labeled cells was recorded and analyzed using a FACS Calibur flow cytometer (Becton Dickinson). Lymphocytes were gated based on forward and sideward scatter (FSC and SSC). CD25^+^Foxp3^+^ conventional Treg cells were determined as the proportion of CD4^+^ T cells. The ratio of CD8^+^ Treg cells was determined as CD25^+^Foxp3^+^ cells in the CD8^+^ lymphocyte gate. To determine the activated and naive/memory T-cell ratio, the following antihuman monoclonal antibodies were used: anti-CD3-FITC, anti-CD8-PE, anti-CD4-PerCP, anti-CD56-PECy5, anti-CD25-PE, anti-CD45RA-PE, and anti-CD45RO-PerCP (all from BD Biosciences). At least 10,000 cells were collected in the lymphocyte gate and analyzed. CD3^+^, CD4^+^, CD8^+^ T cells, CD3^+^CD4^+^CD25^−^ “resting T-helper cells,” and CD3^+^CD4^+^CD25low^+^ “activated” T cells were detected, and their absolute cell numbers were calculated. We tracked the percentages and absolute cell counts of naive CD3^+^CD45RA^+^ and CD3^+^CD45RO^+^ memory T cells.

### Tests of the humoral immune responses

Disease-specific autoantibodies were measured using conventional ELISA tests or by immunoblotting. Systemic and organ-specific autoantibody screening tests (ANA screen Werfen) (ENA screen, anti-phospholipid autoantibody screen, ANCA screen, tTG screen Orgentec) and antigen-specific (TG, TPO, tTG, IA2, GAD65, ASCA, GBM) ELISA tests (Orgentec) were used to detect autoantibodies. Anti-measles antibody (IgG) measurements were performed using a self-developed ELISA assay, as previously reported (18). The anti-measles, anti-mumps, and anti-rubella indirect ELISA IgG ready-to-use kits (Euroimmun, Lübeck, Germany) were used to detect humoral antibody levels according to manufacturer’s instructions, as long-lasting postvaccination immune response. The Mantoux tuberculin skin test was used to determine cell-mediated immunity to *Mycobacterium tuberculosis*. The anti-rubella antibody levels were measured using the previously described ELISA assay (19).

### Statistical analysis

The differences were analyzed by two-way ANOVA with Graph-Pad Prism v8. Data were presented as the mean ± SEM. The difference was considered statistically significant at *p* < 0.05.

## Results

### Clinical characteristics of the patients

**Figure 1:**
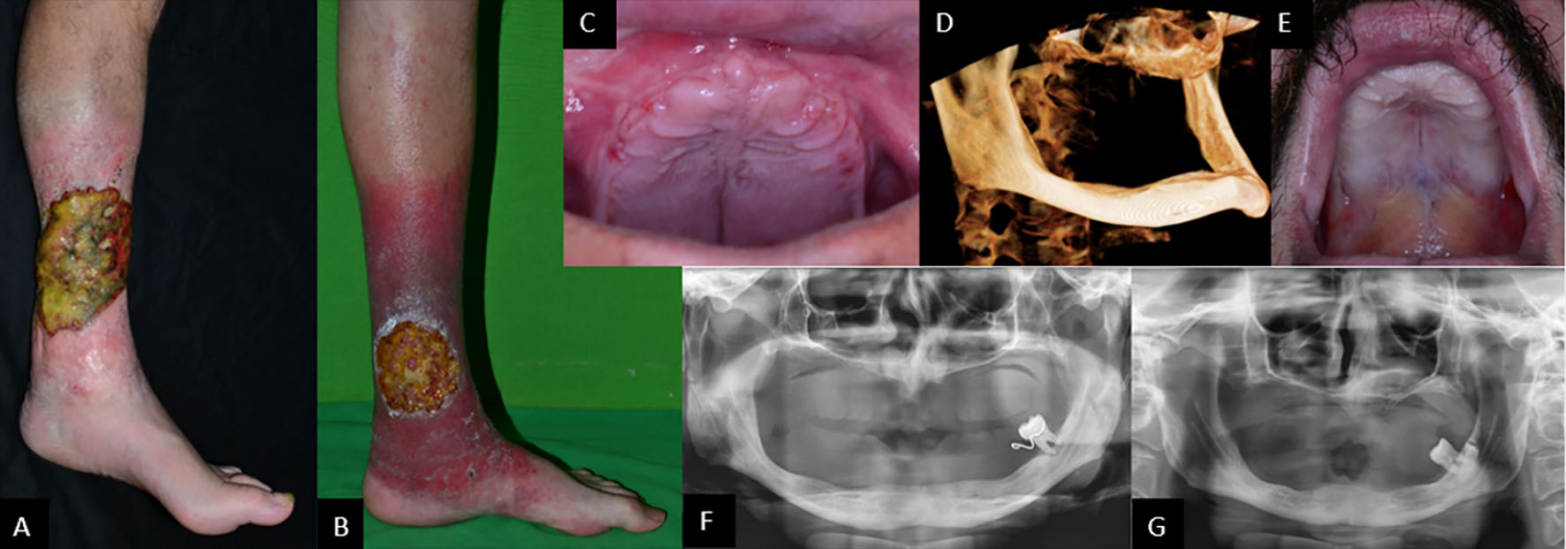
Biofilm-covered, 14-×-18-cm large ulcer in P1 **(A)** and 10-cm-diameter ulcer in P2 **(B)**, both on left leg. Complete tooth loss in P1 **(C)** and P2 **(E)**. Cone-beam CT scan showing severely atrophied jaws and missing processus alveolaris **(D)**. Panoramic radiographs showing highly atrophied jaws and a single remaining erupted tooth in both patients: P1 **(F)** and P2 **(G)**.

Two male siblings (P1: 36 years old and P2: 31 years old) were referred to the Department of Dermatology, Venerology, and Oncodermatology of the University of Pécs because of chronic ulcers on their legs (**Figures 1A, B**). In the case of P1, the ulcers appeared at 17 years of age on the left leg and at 24 years of age on the right leg, while in P2, the age of ulcer onset was 24 years on both lower extremities. In both patients, cellulitis developed after the onset of ulcers. Their family history was negative. Perinatal anamnesis was without any documented alterations in both patients. P1 had recurrent cases of pneumonia between the ages of 4 and 10 years and suffered from thrombophlebitis of the left lower extremity in young adulthood. P2 had recurrent otitis media at age 4–5 years, resulting in tympanic perforation and permanent hearing loss of the right ear. Both siblings had chickenpox, but not herpes zoster. Both patients experienced recurrent swelling and bleeding of the gingiva from age 12. Without any visits to the general dental practitioner, there was no possibility to refer patients to a periodontist specialist. Gradually, periodontitis caused severe generalized alveolar bone loss and resulted in extremely mobile teeth when patients became ~19–20 years old (**Figures 1C–G**). At this stage, patients still had not visited a periodontist. The general dental practitioner made extractions, and the subtotal partially edentulous status was treated with removable prostheses. When further teeth became mobile, only extraction and prosthesis correction were performed. Parents and other known relatives of the patients did not present any of the above symptoms.

**Figure 2:**
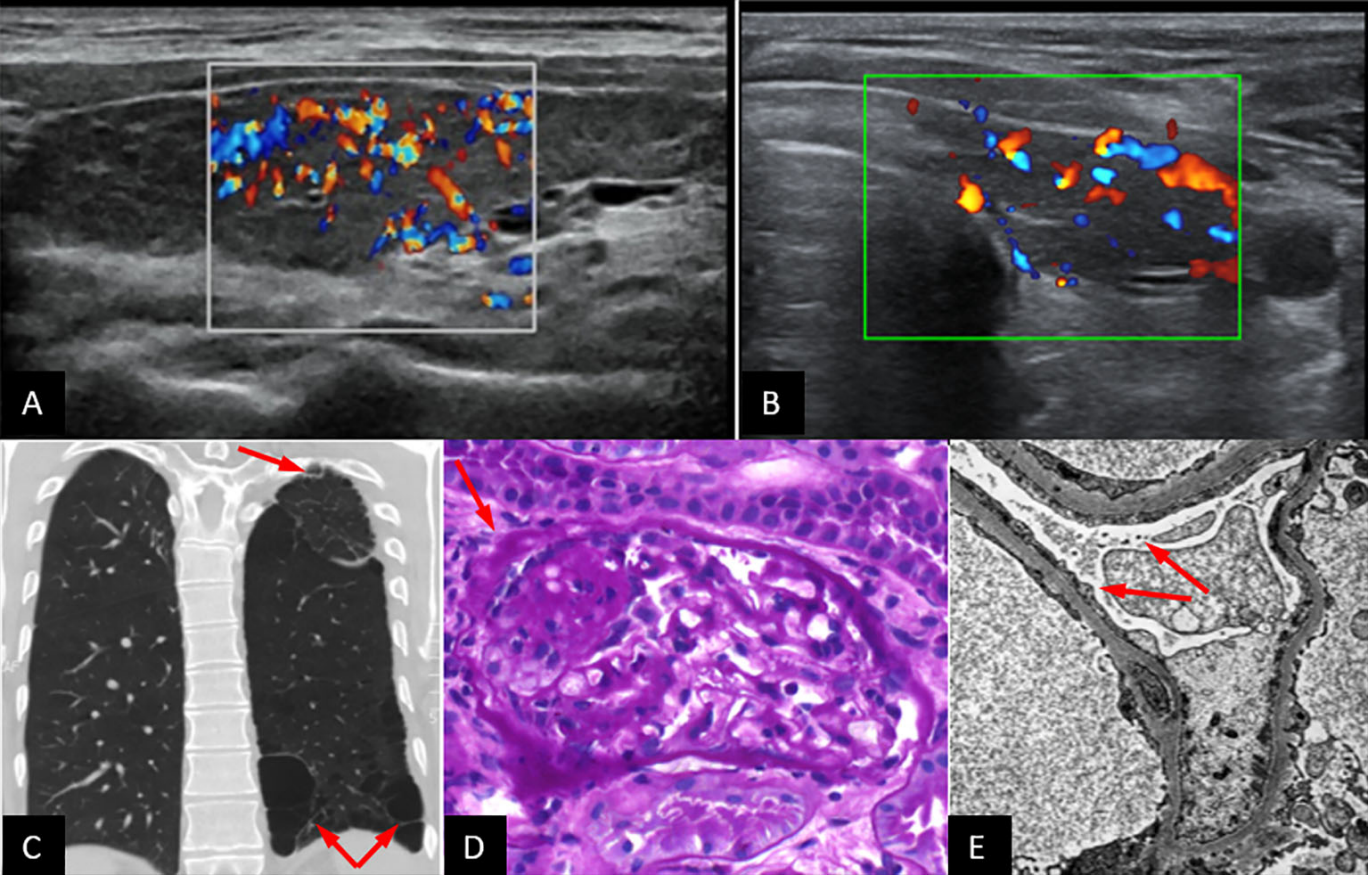
Thyroid ultrasound showing normal-sized, inhomogeneously structured, hypervascularized thyroid and connective tissue alterations, typical signs of Hashimoto’s thyroiditis, in P1 **(A)** and P2 **(B)**. Chest CT of P1. Arrows indicate basal paraseptal bullae, paraseptal emphysema cranially, and dextrally. Apical callus and spread micronodules **(C)**. Kidney biopsy, PAS staining: a sclerotized region with scarring in the glomerulus (red arrow) **(D)**. Kidney biopsy, electron microscopy: podocyte foot processes fused (red arrows) and flattened in 85%–90%, endothelium properly fenestrated. Immune fluorescence did not detect immune complex deposition **(E)**. Diagnosis: focal segmental glomerulosclerosis.

On examination, the patients were of normal height and weight (P1: 76 kg, 182 cm; P2: 64 kg, 183 cm). Routine laboratory tests indicated impaired liver functions and proteinuria in P1, diabetes mellitus in P2, as well as hypothyroidism in both (**Table 1**). A thyroid ultrasound (US) indicated signs of Hashimoto’s thyroiditis (**Figures 2A, B**). In P1 bilateral pleural callus and pulmonary emphysema, enlargement and parenchymal thickening of both kidneys, low-grade hepatomegaly and multiple accessory spleen, and lack of thymus gland were detected by imaging examinations (chest X-ray, CT scans, and abdominal US) (**Figure 2C**).

**Table 1:** Routine laboratory tests, immunohematology I (H, high; L, low).

	P1	P2	Units	Ref. interval
Glucose	5.64	**8.00H**	mmol/L	3.90–6.00
Fructosamine	*x*	**356H**	qmol/L	200–285
Haemoglobin a1c	5.30	**6.80H**	%	4.00–5.60
TSH	**22.59H**	**33.54H**	mU/L	0.270–4.20
Free T4 (fT4)	**8.1L**	**9.95L**	pmol/L	12.0–22.0
Anti-TG	**948.9H**	**337.1H**	IU/ml	<150
Anti-TPO	**214.2H**	**175.8H**	IU/ml	<75.0
Parathormon	3.40	3.90	pmol/L	1.60–6.10
Vitamin 25-OH D3	**10.0L**	**42.4L**	nmol/L	47.7–144
Calcium	**1.99L**	2.36	mmol/L	2.15–2.55
Anorg. phosphat	1.25	1.05	mmol/L	0.81–1.45
GOT	27	17	U/L	<44
GPT	20	16	U/L	<50
GGT	**197H**	23	U/L	<60
Total protein	**39.9L**	77.4	g/L	66.0–87.0
Albumin	**10.2L**	47.4	g/L	35.0–52.0
Carbamide	4.5	5.4	mmol/L	2.14–8.21
Creatinine	56	74	qmol/L	62–106
Urine total protein	**11.84H**	0.09	g/L	<0.10
Urine microalbumin	**8.328H**	9	mg/L	<20
IgA	3.02	4.0	g/L	0.70–4.00
IgM	0.4	0.94	g/L	0.40–2.30
IgG	17.40	14.30	g/L	7.00–16.00
IgG1	7.75	8.96	g/L	4.90–11.40
IgG2	5.51	3.36	g/L	1.50–6.40
IgG3	0.56	0.47	g/L	0.20–1.10
IgG4	0.55	0.52	g/L	0.08–1.40
Lupus anticoagulant test	**+**	**+**		
Lupus anticoagulant	**1.4H** **1.26H**	**1.3H** **1.29H**		0.8–1.2
Antithrombin activity	**150H**	**130H**	%	83–128
Protein C activity	**154H**	**159H**	%	70–130
Protein S activity	99	118	%	64–149
Cryoglobulin serum	**Mildly +**	Negative		
Cryoglobulin citrát	**Mildly +**	Negative		
Cryoglobulin heparin	**Mildly +**	**+**		
Red cell antibody	**Cold antibody**	**Cold antibody**		
Cold agglutinin	**At +4°C (+++)**	**At +4°C (+++)**		

Bold highlighting represents high (H) and low (L) values.

Microscopic examination of kidney biopsy specimen PAS sections from P1 showed glomerular sclerotizing and scarring (**Figure 2D**). Immune fluorescence examination of a kidney biopsy did not detect immune complex deposition. Electron microscopy, however, showed fused and flattened podocyte foot processes in 85%–90% with properly fenestrated endothelium (**Figure 2E**). Together, these findings were consistent with the diagnosis of focal segmental glomerulosclerosis (FSGS). None of these symptoms were detected in P2.

Lower extremity arterial pulses were palpable in both individuals except for the left dorsal pedal artery of both patients and the tibial posterior artery of P2, probably due to ankle swelling distal to the wound. A handheld Doppler signal could be detected over distal arteries. Large arterial stenosis was ruled out by CT angiography. Angiological examinations showed satisfactory lower extremity venous circulation; however, laser Doppler flowmetry detected severely impaired microcirculation in both patients. Transcutaneous tissue oxygen pressure (tcpO2) after a 10-min resting period: P1: left leg 24 mmHg, left foot 26 mmHg; P2: left leg 18 mmHg, left foot: 2 mmHg. Laser Doppler flowmetry showed low values distal to the wound P1: 32°C: 22–26 PU, 44°C: 29–32 PU; P2: 32°C: 18–23 PU, 44°C: 120–140 PU. Polyneuropathy was not suspected by the normal result of the tuning fork vibration test in both patients.

Direct immunofluorescence examination of biopsies from the periphery of ulcers did not show specific immune-reactant deposition or vasculitis. A direct mutation analysis of the complement component 1 subcomponent R and subcomponent S genes (C1R and C1S) (20), responsible for the development of Ehlers–Danlos syndrome, periodontal type 1 (also known as Ehlers–Danlos syndrome type VIII) and Ehlers–Danlos syndrome, periodontal type, 2, respectively, was performed, and no mutation was found. Disease-specific collagen alterations were not detected by electron microscopy.

### Immunoserological parameters

ANA screen, ENA screen, MPO (p-ANCA), Hs-PR3 (c-ANCA), cardiolipin IgG/IgM, B2-glikoprotein IgG/IgM, and prothrombin IgG/IgM were all negative in both patients. HIV-1/HIV-2 antibody, HIV-1 Ag, HBsAg, and HCV Ag were also negative. For P2: significant antibody titer (>100 IU/L, 10.6 mNe/ml) for hepatitis B virus (HBV) anti-HBs (surface) antigen with ELISA. Varicella-zoster (VZV/HH3) IgG was detected *via* ELISA. Complement activity was within reference values; prothrombin INR, thrombin time, D-dimer, fibrinogen, and protein S were also normal in both cases.

### Identification of a hemizygous nonsense variant in the MSN gene through WES

**Figure 3:**
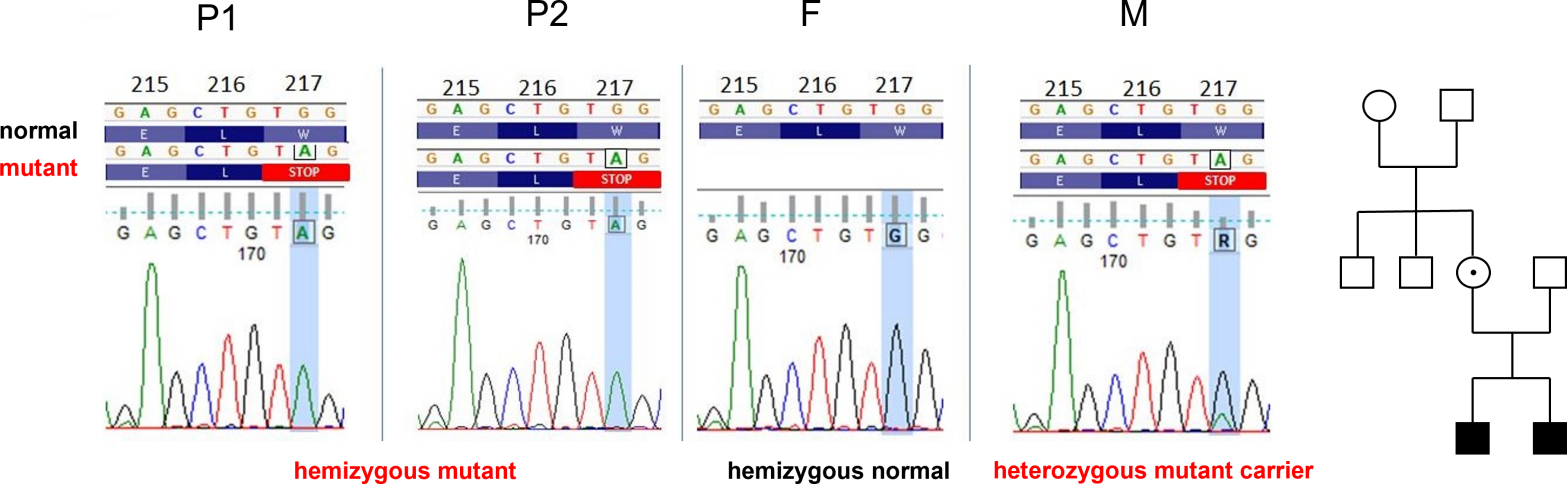
Identical *MSN* gene variants in P1 and P2: c.650G>A p.Trp217Ter, W217X (sixth exon). The father **(F)**, maternal grandmother, and uncles carry wild-type alleles. The mother **(M)** is heterozygote. Family tree: white, wild-type; dotted, heterozygote, black, hemizygote.

To dissect the genetic background of the observed clinical alterations, bioinformatic analysis of the whole exome sequencing (WES) data generated using genomic DNA from P1 was carried out, and a novel hemizygous nonsense mutation, 650G>A in exon 6 of the moesin (*MSN*) gene on chromosome X, was identified. This mutation has not been documented before in the gnomAD, ClinVar, and the HGMD databases. Next, P2, the parents and the mother’s two healthy brothers were directly tested for the identified moesin variant by Sanger sequencing. The same mutation was validated in P2, while in the patient’s mother, heterozygous status was identified. None of the other screened family members carried the mutation (**Figure 3**).

This new mutation detected in the MSN gene is considered to be a variant of uncertain significance (VUS) due to the lack of literature data, but its pathogenicity was assumed with its predicted effect (premature stop codon formation) and the segregation results.

### Dysregulated immunophenotype in patients carrying the novel hemizygous MSN mutation

Since mutations in the MSN gene are known to be associated with immunodeficiency, and since several episodes of recurrent infections occurred in both patients, we further investigated immune function abnormalities. To this end, immunologic profiling was performed using peripheral blood flow cytometry analysis at three different time points (in 2015, 2019, and 2020) (**Table 2**). Although mild anemia was detected in both patients, white blood cells, lymphocyte, neutrophil, eosinophil, and basophil counts were normal, and blood cell morphological abnormalities were not detected. CD3^+^, CD4^+^, CD8^+^ T, and CD19^+^ B-lymphocyte counts and ratios were mostly normal or occasionally slightly outside of the normal ranges. CD56^+^ NK cell ratios, however, were markedly lower than normal in P1. Flow cytometry analysis showed immunophenotypes consistent with immune dysregulation. Naive (CD45RA^+^) T-cell ratios were markedly lower than normal at every measurement in both patients (CD3^+^CD45RA^+^ ratio: P1: 22.1%–27.8%; P2: 17.2%–19.2%; normal range: 33.0%–66.0%). A large proportion of the total T-cell population was CD25^+^, indicating an activated phenotype (CD3^+^CD25^+^ ratio: P1: 21.5%–28.5%; P2: 20.1%–35.7%; normal range: 1.9%–7.7%). Furthermore, more than half of the T-helper population was also activated (CD4^+^CD25^+^ ratio: P1: 53.7%–56.7%; P2: 51.1%–60.4%; normal range: 4.0%–11.0%). Interestingly, the ratio of cells expressing HLA-DR, another indicator of T-cell activation, was found normal within the total CD3^+^ population in P1 on two of three occasions, and in P2 at all three measurements. On the other hand, the CD8^+^HLA-DR^+^ ratio was markedly reduced within the cytotoxic T-cell population (CD8^+^HLA-DR^+^: P1: 1.9%–3.0%; P2: 1.1%–5.7%; normal range: 5.0%–25.0%). The proportion of regulatory T cells, both within the CD4^+^ and the CD8^+^ population, was found normal. CD4^+^/CD25^+^/Foxp3 staining was carried out to identify Treg cell ratios. For P1, CD4^+^ T-helper cells are CD4^+^/CD25^+^/Foxp3^+^ in 3.1%, within the normal range, while CD8^+^ T cytotoxic T cells are CD8^+^/CD25^+^/Foxp3^+^ in 0.1%, similar as compared to healthy controls. For P2, the Treg ratio is 5.7% and CD8^+^ cytotoxic T cells are CD8^+^/CD25^+^/Foxp3^+^ in 0.8% (**Table 2**). Taken together, these results indicate that the novel *MSN* variant leads to impaired lymphocyte functions, primarily affecting T cells. A low CD8^+^ cell ratio and absolute cell count indicate a disturbance in cell activation. This is accompanied by a low naive T-cell ratio, which supports a maturational defect in T cells, and may indicate congenital immunodeficiency.

**Table 2:** Hematology, immunohematology II, and FOXP3 staining results (ND, not determined; H, high; L, low).

Hematology		P1			P2		Units	Ref. interval
**Year of evaluation**	**2015**	**2019**	**2020**	**2015**	**2019**	**2020**		
Hemoglobin	**111L**	**118L**	**121L**	**123L**	**116L**	**117L**	g/L	137–175
Hematocrit	**32.1L**	**36.7L**	**36.0L**	**35.5L**	**37.1L**	**36.0L**	**%**	40.1–51.0
Red cell count	**3.90L**	**4.06L**	**4.09L**	**4.13L**	**3.88L**	**3.85L**	T/L	4.50–6.00
White blood cell count	5.17	5.85	8.01	6.57	8.25	7.56	Giga/L	4.00–10.00
Neutrophils	2.47	2.7	4.65	3.70	5.03	3.70	Giga/L	1.78-5.38
Lymphocytes	1.57	2.08	1.71	1.62	2.12	2.43	Giga/L	1.32–3.57
Monocytes	0.620	**0.90H**	**1.29H**	0.670	**0.91H**	**1.17H**	Giga/L	0.30–0.82
Eosinophils	0.180	0.14	0.29	0.190	0.110	0.19	Giga/L	0.00–0.54
Basophils	0.020	0.01	0.02	0.020	0.020	2.02	Giga/L	0.00–0.08
Platelets	173.0	221.0	205.0	194.0	225.0	192	Giga/L	140.0–440.0
∑ Lymphocytes	1,570	2,080	1,710	1,620	2,120	**2,430H**	/ql	1,200–2,400
CD3^+^ T-cell ratio	78.7	72.3	79.5	69.1	69.8	**61.1L**	%	64.0–82.0
∑ CD3^+^ T cells	1,236	1,504	1,359	1,119	1,480	1,485	/ql	984–1,984
∑ CD4^+^ T cells	**510L**	763	**593L**	748	1039	858	/ql	643–1,175
∑ CD8^+^ T cells	595	666	576	**334L**	363	532	/ql	336–876
CD4^+^ T-cell ratio	**32.5L**	36.7	**34.7L**	46.2	49.0	**35.3L**	%	36.0–54.0
CD8^+^ T-cell ratio	**37.9H**	32.0	33.7	**20.6L**	**17.1L**	**21.9L**	%	22.0–36.0
CD4/CD8 ratio	**0.86L**	1.15	1.03	2.24	2.87	1.61		0.92–4.11
CD19^+^ B lymphocytes	16.0	**21.5H**	13.8	10.6	11.5	14.2	%	7.2–16.4
∑ CD19^+^ lymphocytes	251	**447H**	236	172	244	345	/ql	97–399
CD5^+^ B lymphocytes	2.6	4.6	2.0	1.3	1.6	1.9	%	<5.0
∑ CD5^+^ B lymphocytes	41	96	34	21	34	46	/ql	
CD56^+^ NK cells	**4.2L**	**3.8L**	**5.6L**	16.1	15.3	25.4	%	9.6–27.0
CD3^+^, CD56^+^ NKT cells	18.1	1.7	2.2	4.0	1.5	4.9	%	
**T-cell subpopulations**								
CD3/CD45RA^+^ (naive)	**26.5L**	**22.1L**	**27.8L**	**19,2L**	**18.7L**	**17.2L**	%	33.0–66.0
CD3/CD45RO^+^ (memory)	55.9	56.4	52.2	53.3	49.2	44.5	%	24.0–57.0
CD3/CD25^+^ (activated)	**21.5H**	**24.1H**	**28.5H**	**35.7H**	**30.1H**	**20.1H**	%	1.9–7.7
CD4+CD25^+^ (activated) Th	**56.0H**	**53.7H**	**56.7H**	**60.4H**	**59.5H**	**51/1H**	%	4.0–11.0
HLA-DR/CD3^+^ activated	6.6	**1.9L**	4.0	7.5	4.3	2.8	%	2.4–11.8
HLA-DR/CD8^+^ activated	**2.6L**	**1.9L**	**3.0L**	5.7	**1.6L**	**1.1L**	%	5.0–25.0
CD4^+^CD25high (Treg)	2.4	2.5	4.7	6.4	2.7	4.4	%	
CD4^+^CD25^+^Foxp3^+^ (Treg)	ND	ND	3.1	ND	ND	5.7	%	
CD8^+^CD25^+^Foxp3^+^ (Treg)	ND	ND	0.1	ND	ND	0.8	%	
Th1/Th2 intracellular cytokines								
Interleukin-4%				1.00	ND		%	
Interferon-gamma %				3.00	ND		%	

Bold highlighting represents high (H) and low (L) values.

### The novel MSN variant leads to diminished moesin mRNA and protein expression

**Figure 4:**
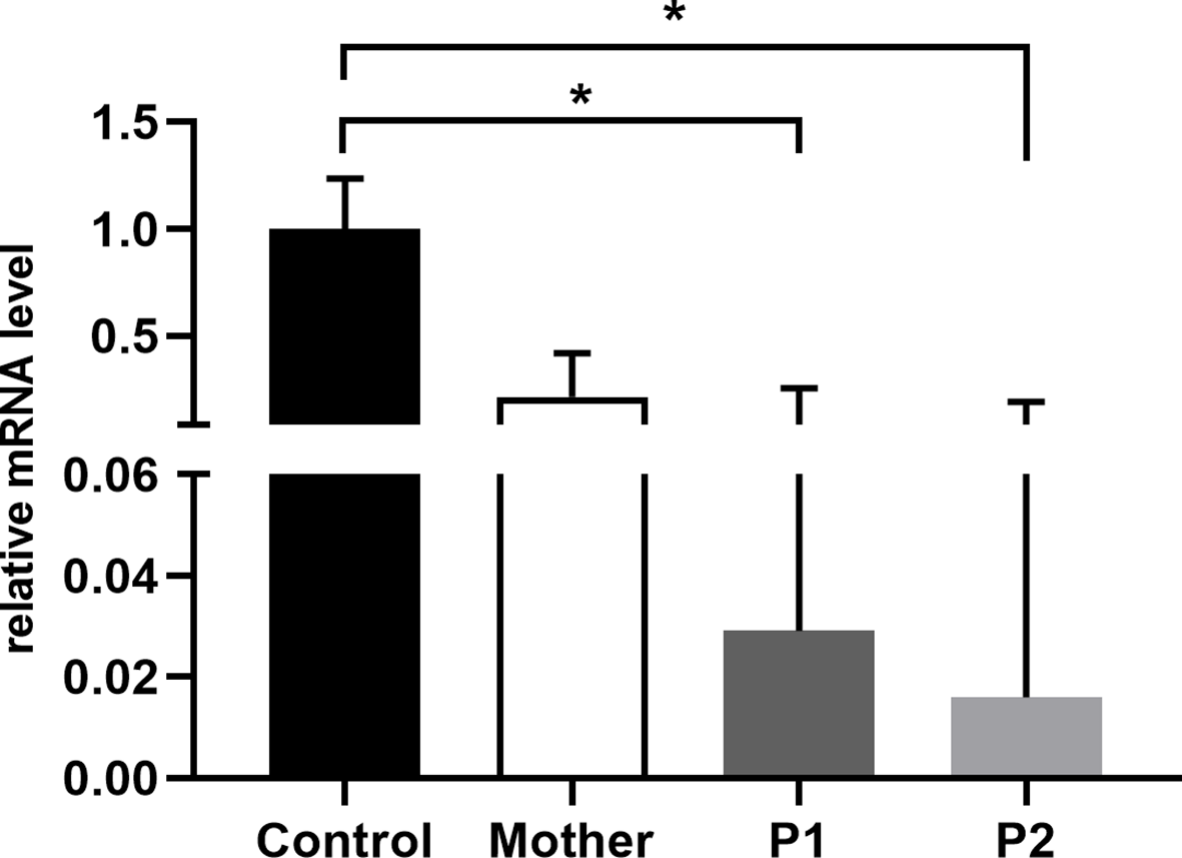
Relative quantity of MSN mRNA in PBMCs of healthy controls, mothers, and patients. Data are mean ± SEM for *n* = 4/healthy control and triplicates/mother, patients. ^*^ *p* < 0.05 control vs. mother, P1, and P2, based on one-way ANOVA followed by Bonferroni’s *post-hoc* test.

**Figure 5:**
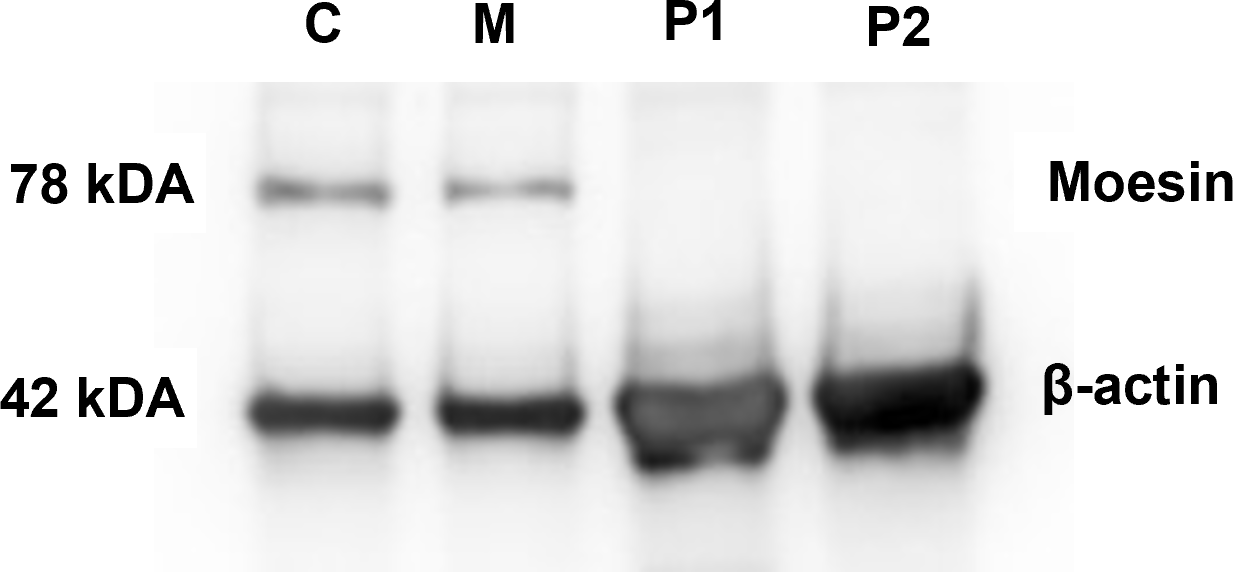
MSN protein detection. Western blot analysis showing the presence of moesin in PMBCs of healthy controls **(C)** and the patients’ mothers **(M)**. Beta-actin was used as the loading control.

Moesin mRNA expression in PBMCs of the patients, the mother, and healthy controls was analyzed by qPCR. Compared to the controls, significantly decreased MSN transcript values were detected in the patients and lower mRNA expression in the mother. The decreased expression in the mother could be the consequence of the heterozygous mutation in the MSN gene (**Figure 4**). As the previously documented *MSN* mutations were associated with altered MSN protein expression, we hypothesized that the novel mutation might affect the expression of the MSN protein as well. A Western blot analysis was performed using an antibody directed against the full-length MSN protein (clone MSN/491). PMBCs of a healthy volunteer and the mother expressed the full-length moesin protein in a significant amount. The truncated 216 amino acid protein has a predicted 25-kDa molecular weight; however, this could not be detected in the patient’s samples with the MSN antibody used (**Figure 5**). Transcripts with early stop codon are recognized and eliminated *via* nonsense-mediated mRNA decay (NMD) to prevent the accumulation of truncated proteins (21, 22). Further examinations are needed to confirm or exclude the NMD processes and to detect the presence of the truncated protein.

### The novel MSN variant may lead to impaired T-cell proliferation

**Figure 6:**
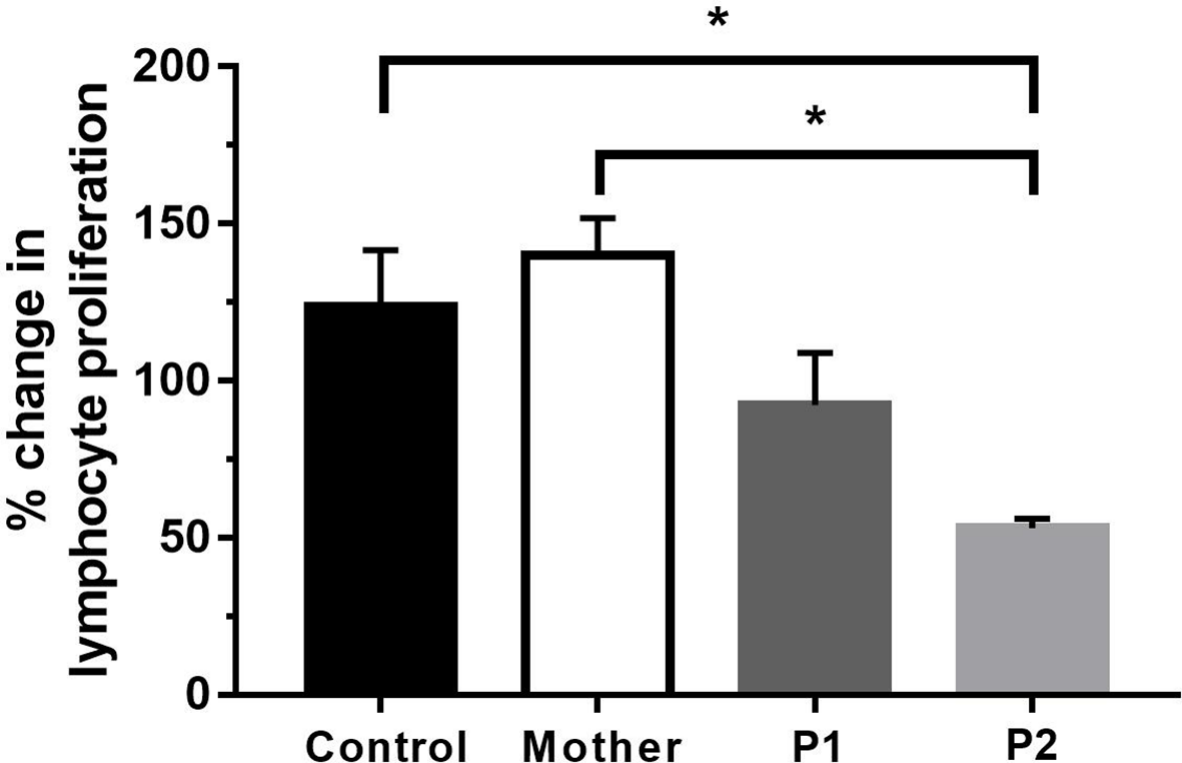
Proliferation of lymphocytes after 72 h of PHA activation. Data are the mean ± SEM of three independent experiments. Control represents the results of six healthy volunteers. ^*^ *p* < 0.05 healthy control and mother vs. P2, based on one-way ANOVA followed by Bonferroni’s *post-hoc* test.

Since we detected profound immunophenotype alterations in our patients, we then wanted to test the functional capacity of T lymphocytes. The PHA-induced proliferative response of peripheral mononuclear cells was compared between the patients, their mother, and the healthy controls. Proliferative responses of P2 (53.1 ± 3.024) were significantly decreased compared to those of the healthy volunteers (123.7 ± 17.86) and the mother (140.1 ± 11.64). In the case of P1, the PHA-induced proliferation was somewhat lower, but the difference was not significant between healthy volunteers and the mother (**Figure 6**). The Tuberculin Skin Test (Mantoux test) showed hyperergic reaction in P1 (NB: as a result of compulsory BCG vaccination in Hungary, individuals with normal immune functions display hyperergic Mantoux test—a sign of type IV reaction against mycobacterial purified protein derivative). P2 refused the Mantoux test. The Quantiferon-TB test was negative in both P1 and P2.

### Dysregulated B-cell functions in patients with novel MSN mutation

**Figure 7:**
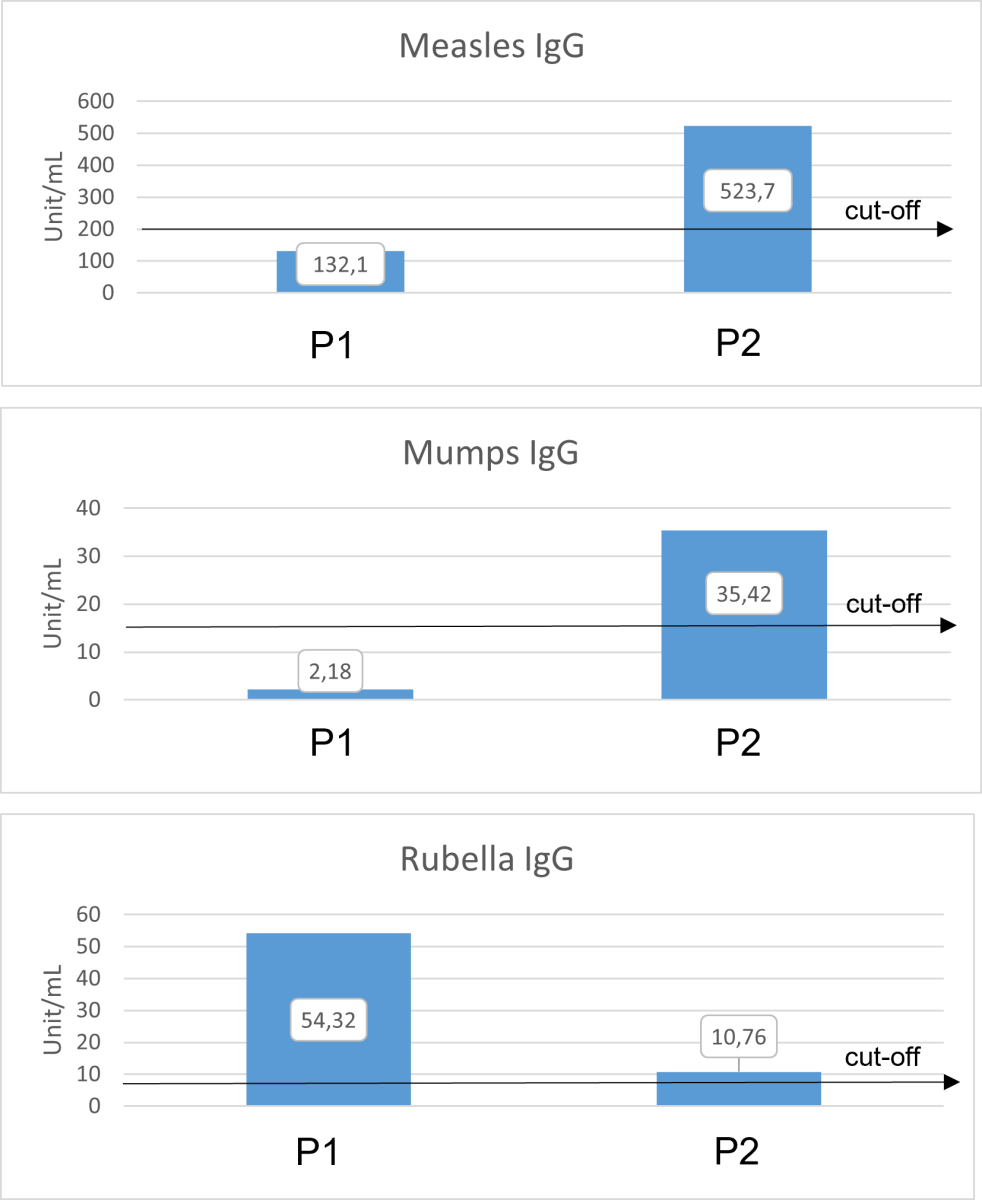
Anti-measles, anti-mumps, and anti-rubella antibody titers (Euroiummun, IgG) compared to the relative cutoff values. Anti-measles IgG cutoff = 200 U/ml. Anti-measles IgG P1 = 132.18 U/ml (negative), P2 = 523.77 U/ml (positive). Anti-mumps IgG cutoff = 16 U/ml. Anti-mumps IgG P1 = 2.18 U/ml (negative), P2 = 35.42 U/ml (positive). Anti-rubella IgG cutoff = 8 U/ml. Anti-rubella IgG P1 = 54.32 U/ml (positive), P2 = 10.76 U/ml (positive). Black arrows show cutoff values.

Both patients had elevated antithyroglobulin (anti-TG) and antithyroperoxidase (anti-TPO) antibody titers and were positive for lupus anticoagulant, cryoglobulin (heparin), red cell antibody, and cold agglutinin (reacted at +4°C) and had elevated protein C and antithrombin activity (**Table 1**). Since live attenuated vaccines (LAVs) stimulate a strong, effective, and long-lasting immune response, we used anti-measles, anti-mumps, and anti-rubella (MMR LAV) postvaccination humoral antibody (IgG) titer testing as a model to investigate potentially impaired B-cell functioning. According to their vaccination certificates, P1 received primary and reminder measles vaccines at ages 14 months and 11 years, while P2 was vaccinated against measles and rubella at age 14 months and against measles, mumps, and rubella at 11 years (**Table 3**). Anti-measles, anti-mumps, and anti-rubella IgG antibody titers are presented in (**Figure 7**). In the case of P1, we detected insufficient circulating IgG antibody levels for measles and mumps (measured value/cutoff values were 132.18/200.0, 2.18/16.0, and 54.32/8.0 U/ml for measles, mumps, and rubella, respectively). In the case of P2, we detected sufficient antibody titers for all vaccines (measured value/cutoff values were 523.77/200.0, 35.42/16.0, and 10.76/8.0 U/ml for measles, mumps, and rubella, respectively, according to the International Notes, Hungary) (23).

**Table 3:** Patients’ data and qualitative MMR ELISA (IgG) results.

	Date of birth	Immunization I.	Immunization II.	Quantitative ELISA results (Euroimmun, IgG)		
				Measles	Mumps	Rubella
**P1**	1,984.06.12	1,985.08.05. (Morbilli)	1,996.11.19. (Morbilli)	Negative	Negative	Positive
**P2**	1,989.04.18	1,990.06.18. (Morbilli-rubella)	2,001.10.16. (MMR)	Positive	Positive	Positive

Vaccination certificates registered immunization data of patients versus MMR vaccine-induced, measured qualitative results of humoral antibody (ELISA, IgG) levels.

### Therapy and clinical outcome

There are currently no approved therapeutic recommendations in IMD50. The siblings did not require intravenous immunoglobulin therapy or granulocyte colony-stimulating factor. They were in satisfactory general health conditions at the time of the writing of the manuscript.

Angiological examinations detected severely impaired microcirculation in both patients. Due to the antiphospholipid syndrome, hematology suggested the introduction of anticoagulant vitamin K-antagonist therapy, which was not tolerated by P1 and refused by P2. Low molecular weight heparin therapy completely resolved ulcers in P1 in 6 months, while P2 refused this as well. In P1, noninvasive control studies in angiology showed a significant improvement in microcirculation. Transcutaneous tissue oxygen pressures (tcPO_2_) on the left foot were measured after ulcer healing with the following tension values: 72 mmHg after 15 min resting period; 74 mmHg 5 min, 84 mmHg 10 min, and 89 mmHg 15 min after the walk test. The laser Doppler flowmetry measurements on the left big toe showed 135 PU on average and 122 PU after the walk test (pavement test at 10% gradient, 3.2 km/h, 5 min). P2 refused follow-up testing. No abnormalities suggestive of lower limb arterial circulatory dysfunction were detected at rest and were not provoked by exercise. Wound healing is explained by the microcirculation-improving effect of anticoagulant treatment. Local antiseptic treatment and antimicrobial foam dressing appropriate to the wound condition were applied, but no treatment modification was made.

Hormone replacement therapy in P1 leads to normalized thyroid hormone levels. P2 refused any thyroid hormone therapy. FSGS in P1 improved upon conservative and systemic glucocorticoid treatment, with less than 0.5 g/day proteinuria following 6 months of treatment, and upheld glomerular function throughout.

Both patients had first partial removable and later total removable dental prostheses. Implantation therapy was not a real alternative partly because of the family’s financial circumstances and partly because of the highly questionable outcome.

## Discussion

Here, we describe a pair of male siblings with a novel autoimmune phenotype, namely antiphospholipid syndrome, in connection with Immunodeficiency 50. We identified a hemizygous nonsense mutation of the *MSN* gene (p.W217X) as the genetic alteration responsible for the clinical findings. The newly identified mutation was different from the previously documented cases of *MSN* mutations (**Table 4**). In eight of those cases, an identical missense mutation (c.511C>T) was identified, leading to an arginine-to-tryptophan transition at an amino acid position of 171 (R171W). The remaining case carried a nonsense mutation (c.1657C>T), generating a premature stop codon at position 553 (R533X) (10–12). The newly identified mutation also leads to the formation of a premature stop codon at amino acid 216 (p.Trp217Ter) and a significantly truncated and likely dysfunctional MSN protein (the full-length moesin protein consists of 577 amino acids).

**Table 4:** Clinical and laboratory comparison of previously studied MSN deficiencies (PP1–PP9) vs. current subjects (P1 and P2).

Case	MSN mutation	Bacterial infections	Varicella-zoster	Eczema	Molluscum contagosum	Autoimmunity	Persistent lymphopenia	Fluctuating neutropenia	IgG therapy	Improvement with G-CSF	Alive and well
PP1	R171W	Yes	Yes	Yes	Yes	No	Yes	Yes	Yes	No G-CSF	Yes
PP2	R171W	Yes	Yes	Yes	Yes	No	Yes	Yes	Yes	Yes	Yes
PP3	R171W	Yes	Yes	No	No	TTP	Yes	Yes	Yes	No G-CSF	Yes
PP4	R171W	Yes	Yes	Yes	No	No	Yes	Yes	Yes	Yes	Yes
PP5	R171W	Yes	No	Yes	No	No	Yes	Yes	Yes	No G-CSF	Yes
PP6	R171W	Yes	Yes	Yes	Yes	No	Yes	Yes	Yes	No-G-CSF	Yes
PP7	R553X	Yes	No	No	No	No	Yes	Yes	Yes	No-GCSF	Yes
PP8	R171W	Yes	Yes	No	No	ITP	Yes	Yes	Yes	Yes	Yes
PP9	R171W	No	No	Yes	No	No	Yes	Yes	Yes	No	Yes
P1	W217X	Yes	No	No	No	HT, LA	No	No	No	No	Yes
P2	W217X	Yes	No	No	No	HT, LA	No	No	No	No	Yes

PP9: neonatal case. Lymphopenia, neutropenia, varicella-zoster, and molluscum contagiosum did not occur in the studied patients. TTP, thrombotic thrombocytopenic purpura; ITP, idiopathic thrombocytopenic purpura; HT, Hashimoto’s thyroiditis; LA, Lupus anticoagulant.

Since moesin has been shown to have a crucial role in lymphocyte homeostasis, lack or dysfunctionality of moesin protein could account for many or most of the immune abnormalities seen in both our patients and the previously reported cases with MSN mutations. Similar to the previously described R171W and R553X IMD50 variants, recurrent respiratory and ear infections were also observed in our patients with the novel W217X variant. There are, however, significant phenotypic differences between the previously reported cases and our patients, such as the presence of antiphospholipid antibodies, leg ulcers, and significant early tooth loss, or the lack of molluscum contagiosum or recurrent varicella-zoster virus infections in our cases. While the R171W mutation was reportedly associated with autoimmunity in the form of thrombotic thrombocytopenic purpura (TTP) and idiopathic thrombocytopenic purpura (ITP), our patients had organ-specific autoimmunity: Hashimoto’s thyroiditis (HT) and antiphospholipid syndrome with cryoglobulinemia, red cell antibody, and cold agglutinin positivity. Based on the chronic microcirculatory dysfunction and the repeated lupus anticoagulant positivity, the clinical diagnosis of the antiphospholipid syndrome could be established. As moesin has been previously associated with self-tolerance, our clinical observation about this linkage may urge further investigations in this direction (13). To our knowledge, no monogenic cause has been found in connection with antiphospholipid syndrome, but evidence suggests that anti-moesin antibodies may play a role in the pathomechanism of antiphospholipid syndrome (14, 15). It is currently unclear why antiphospholipid antibodies were only observed in our patients and not in those with other MSN mutations. Furthermore, we suggest that the antiphospholipid syndrome-associated microcirculatory dysfunction was the primary causative factor in ulcer formation in our cases. However, it should also be noted that ERM proteins play a central role in endothelial homeostasis and may also be linked to diabetic angiopathy. MSN dysregulation may impair the endothelial barrier, resulting in lymphocyte recruitment and elevated fibronectin expression and deposition in the ECM, which might be a potential mechanism of ulcer formation.

Both panoramic radiographs and cone-beam CT showed severely atrophied jaws in our patients, and the extreme extent of bone loss was very unusual considering the age of the patients. Classical, known syndromes could not be accounted for the loss of teeth. Since dental status suggests severe periodontitis, and moesin is involved in several innate and adaptive immune functions, moesin dysfunction may be indirectly linked to tooth loss. Recognition of lipopolysaccharide (LPS) components of the outer membrane of gram-negative bacteria by monocytes/macrophages is an important step in the immediate and active adaptive immune response (24). It has been reported that LPS stimulation causes increased expression of moesin (25). Moesin, on one hand, can directly bind to LPS, while on the other hand, it can be associated with TLR4 and MD-2. This response involves the activation of inflammatory cytokines (TNF-α and IL-1β) through the activation of a complex network of cytoskeletal proteins, kinases, and transcription factors including CD14; TLR4; MD-2; MAPKs such as ERK1, ERK2, p38 MAPK, and c-Jun N-terminal kinases (JNK); and NF-κB (26, 27). In addition, inhibition of moesin interrupts LPS response pathways through a blockade of MyD88, IRAK, and TRAF6 (28). This hinders the activation of the MAP kinases p38 and ERK and the activation of NF-κB, which regulates TNF-α and IL-1β production (29). Thus, moesin may play an important role in innate immune responses and TLR4-mediated pattern recognition in periodontal diseases (25).

Secondary FSGS in P1 was ruled out by histology and clinical features, suggesting primary, immune-mediated FSGS (30). The effectiveness of glucocorticoids in our case and in many podocytopathies (kidney diseases in which direct or indirect podocyte injury drives proteinuria or nephrotic syndrome) suggests immune system-related pathogenesis of the kidney symptoms (31). This is further supported by the observation that the typical manifestations of podocytopathies on kidney biopsy are minimal change lesions or focal segmental glomerulosclerosis lesions (32, 33). Moreover, patients with podocytopathies may present with alterations in circulating T-cell subsets (e.g., reduced number of regulatory T cells, with normalization when remission is achieved) (34), implying that the MSN mutation-associated immune abnormalities could have had a pathogenetic role in the development of the kidney symptoms in P1. Despite normal levels of immunoglobulins, a potential defect in specific antibody formation could not be ruled out. On the other hand, MSN deficiency-associated direct cytoskeletal function impairments could also have resulted in the aberrant podocyte morphology seen on electron microscopy.

Of note, MSN is also expressed at elevated levels in the lung (**Supplementary Figures S1A, B**), particularly in the distal epithelium (35). This is also suggestive of the role of MSN mutations in recurrent childhood pulmonary diseases and resulting emphysema, potentially through the modulation of alveolar structure and its effect on pulmonary inflammation.

**Figure 8:**
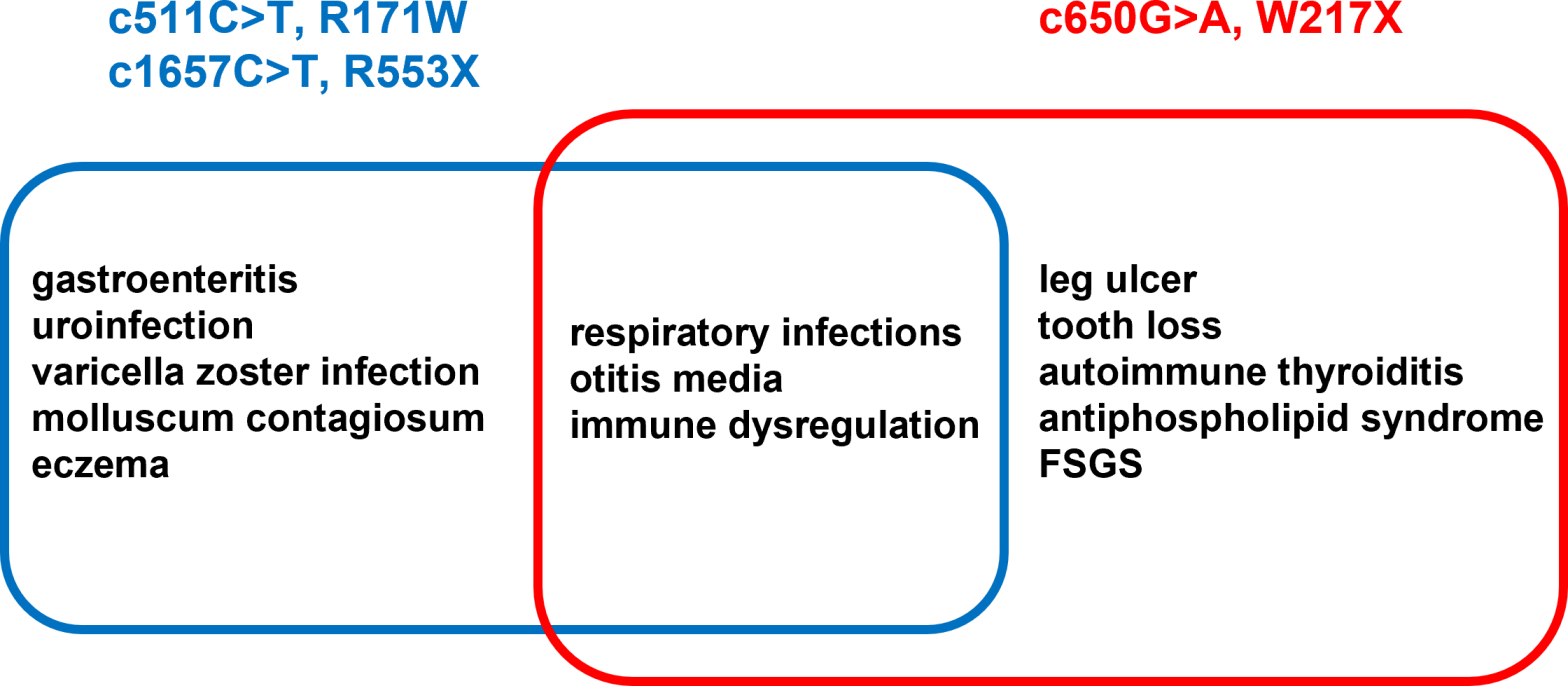
List of concomitant diseases described in eight previous reports (blue) vs. the siblings described in the current work (red).

In conclusion, here, we describe two siblings with a new mutation of the MSN gene, leading to a significantly truncated and likely dysfunctional moesin protein. As the MSN protein plays a wide range of functions in both immune and structural cells, precise identification of its role in symptomatology remains challenging. However, as the dominant factors are immune dysregulation and coupled organ-specific autoimmunity (**Figure 8**), the identified MSN mutation W217X is proposed as a novel autoimmune phenotype of IMD50. Further effort is needed to identify other patients with IMD50 and organ-based autoimmunity to ensure this clinical presentation is part of the spectrum of the disease. So we plan to launch a study on male patients with the autoimmune phenotype (e.g., antiphospholipid syndrome) to check their genetic background with WES and identify probable MSN pathogenic variants.

## Data availability statement

The datasets presented in this study can be found in online repositories. The name of the repository and accession number can be found below: EMBL’s European Bioinformatics Institute; PRJEB52893.

## Ethics statement

Ethical review and approval was not required for the study on human participants in accordance with the local legislation and institutional requirements. The patients/participants provided their written informed consent to participate in this study. Written informed consent was obtained from the individuals for the publication of any potentially identifiable images or data included in this article.

## Author contributions

AK and RG conceived and designed the research. AK, JK, VN, SH, and RG wrote the first draft of the manuscript. AK, ZP, GK, KK, ZN, JSz, TV, KM, TB, KB, CG, JSe, and ME performed functional experiments and analysis and drafted the clinical sections of the manuscript. AK and JK collected the individual clinical data. ZP, DC, and TK performed the sequencing, data analysis, and interpretation and drafted the exome sequencing method and analysis sections of the manuscript. KB, VN, and SH performed experiments and analyzed data. AK, KB, SH, VN, and PO performed the visualization. TK, TB, and RG edited and revised the manuscript. All the authors have read and agreed to the published version of the manuscript.

## Funding

This study was supported by the GINOP-2.3.2-152 grant (TK). Project no. TKP2021-EGA-10 has been implemented with the support provided by the National Research, Development and Innovation Fund of Hungary, financed under the TKP2021-EGA funding scheme (AK, TB, KB, SH, VN, RG). The state funders played no role in the study design, data collection or analysis, decision to publish, or in the preparation of the manuscript.

## Conflict of interest

The authors declare that the research was conducted in the absence of any commercial or financial relationships that could be construed as a potential conflict of interest.

## Publisher’s note

All claims expressed in this article are solely those of the authors and do not necessarily represent those of their affiliated organizations, or those of the publisher, the editors and the reviewers. Any product that may be evaluated in this article, or claim that may be made by its manufacturer, is not guaranteed or endorsed by the publisher.
