# Supplementary material for: Hemizygous nonsense variant in the moesin gene (MSN) leads to a new autoimmune phenotype of Immunodeficiency 50
Source: Front Immunol. 2022 Sep 1;13:919411. doi: 10.3389/fimmu.2022.919411 (PMC9477008; doi:10.3389/fimmu.2022.919411)
Supplement: Supplementary file 1 [file DataSheet_1.pdf]

Supplementary Material

1 Additional radiological examination

DEXA scan indicated osteopenia in P1 (lumbar spine total T score: -2.0; hip T score: -1.6; FRAX index MOF: 1.5%; HF: 0.2%), and osteoporosis in P2 (lumbar spine total T score: -2.5; hip T score: -1.9; FRAX index MOF: 1.2%, HF: 0.3 %.).

CT indicated Riedel lobe in both P1 and P2; heart US did not find abnormalities; heart MRI detected widening of the sinus coronaries (21mm) and persistent left vena cava superior, with normal chamber size and function.

Hepatomegalia and elevated GGT in P1 warranted fibroscan examination, which ruled out hepatic fibrosis, but indicated steatosis (LS: 4,1 kPa, IQR: 0,8, V/IV: 10/0, success: 100 %, METAVIR F0.).

2 Additional clinical examination

Rheumatological examination ruled out chronic joint pain, peripheral joint deformity, hypermobility, kyphosis, scoliosis, synovitis. Connective tissue defects and Sjörgen's syndrome were ruled out. Ophtalmology did not find any indicative symptoms.

3 Laboratory tests

P2: no nephrosis (total protein 0,090 g/l, microalbumin 9 mg/l, kreatinine 8394 umol/l). Serum PTH, FSH, androstendione, testosterone, dehidroepiandroszteron-s, 5-alpha-dihydrotestosterone, prolaktin levels normal in both patients.

A

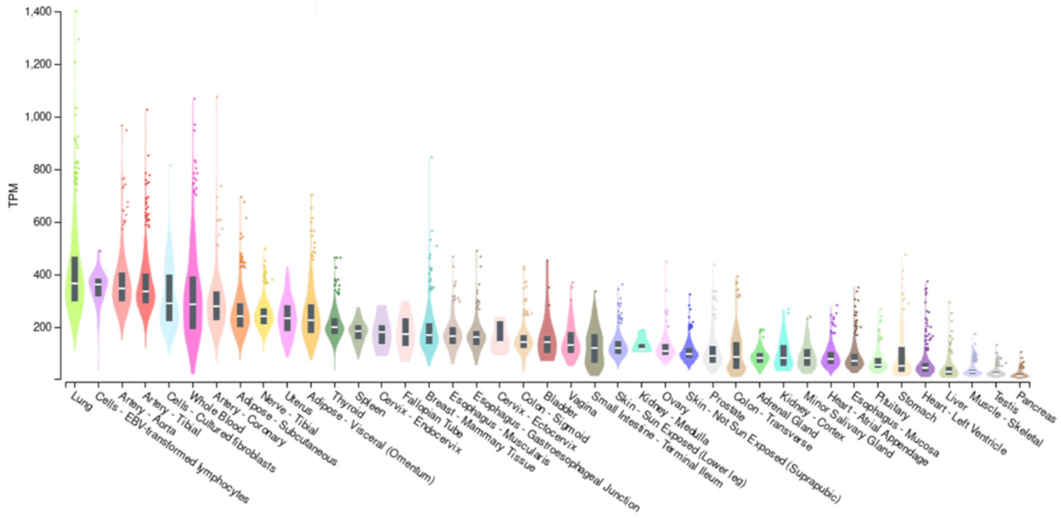

B

Sample cell type / tissue type	MSN mRNA mean expression
Neutrophils, donor3	30,36
Neutrophils, donor2	23,93
lung, right lower lobe, donor1	23,71
Eosinophils, donor3	21,33
Smooth Muscle Cells - Brachiocephalic, donor1	20,29
CD14+ monocytes - treated with Salmonella, donor1	19,81
Neutrophils, donor1	19,10
Endothelial Cells - Aortic, donor3	18,94
breast, adult, donor1	18,85
Endothelial Cells - Aortic, donor0	18,34
Preadipocyte - breast, donor2 (nuclear fraction)	17,57
lymph node, adult, donor1	17,49
Endothelial Cells - Artery, donor3	17,35
Endothelial Cells - Microvascular, donor1	17,22
Smooth Muscle Cells - Umbilical Artery, donor1	17,10
Renal Glomerular Endothelial Cells, donor1	17,06
Fibroblast - skin dystrophia myotonica, donor1 (nuclear fraction)	17,04
Smooth Muscle Cells - Coronary Artery, donor1	16,93
Renal Glomerular Endothelial Cells, donor2	16,80
Eosinophils, donor2	16,48

Supplementary Figure 1. (**A-B**) Expression context of MSN in relevant databases (Gene-Tissue Expression Atlas)
